# Quantification of the Actin-Binding Protein Flightless-I in Human Serum by Automated Western Blot System and Investigation of Its Diagnostic Potential in Sepsis

**DOI:** 10.3390/biomedicines13122850

**Published:** 2025-11-21

**Authors:** Balázs Szirmay, Dániel Ragán, Tamás Huber, Beáta Bugyi, Natália Tóth, László Deres, Diána Mühl, Csaba Csontos, Róbert Halmosi, Attila Miseta, Tamás Kőszegi, Zoltán Horváth-Szalai

**Affiliations:** 1 Department of Laboratory Medicine, University of Pécs Medical School, Ifjúság u. 13, 7624 Pécs, Hungary; ragandaniel@hotmail.com (D.R.); attila.miseta@aok.pte.hu (A.M.); koszegi.tamas@pte.hu (T.K.); horvath-szalai.zoltan@pte.hu (Z.H.-S.); 2 János Szentágothai Research Center, Molecular Medicine Research Group, University of Pécs, Ifjúság u. 20, 7624 Pécs, Hungary; deres.laszlo@pte.hu (L.D.); halmosi.robert@pte.hu (R.H.); 3 Department of Anaesthesiology and Intensive Therapy, University of Pécs Medical School, Ifjúság u. 13, 7624 Pécs, Hungary; toth.natalia2@pte.hu (N.T.); muhl.diana@pte.hu (D.M.); csontos.csaba@pte.hu (C.C.); 4 Department of Biophysics, University of Pécs Medical School, Szigeti u. 12, 7624 Pécs, Hungary; 5 Department of Medical Biology, University of Pécs Medical School, Szigeti u. 12, 7624 Pécs, Hungary; beata.bugyi@aok.pte.hu; 6 1st Department of Internal Medicine, University of Pécs Medical School, Ifjúság u. 13, 7624 Pécs, Hungary

**Keywords:** sepsis, flightless-I, actin-binding proteins, biomarkers, Simple Western

## Abstract

**Background**: The actin-binding protein Flightless-I (Flii) has not been quantified in the human serum yet. We aimed to determine serum Flii levels in healthy individuals and to investigate Flii as a potential marker in patients with sepsis focusing on diagnosis, organ failures, and short-term mortality. **Methods**: A total of 30 controls and 64 septic and 22 non-septic patients were enrolled in this follow-up study. Serum Flii levels were quantified by using the capillary electrophoresis-based Simple Western™ system with chemiluminescent detection. The assay was calibrated by applying dilution series of a purified recombinant human Flii standard and a parallel internal standard. **Results**: Flii levels of healthy controls were found between 3.5 and 8.8 mg/L, while septic and non-septic patients showed significantly lower values (*p* < 0.001). First-day Flii could differentiate sepsis from the non-septic inflammatory state (AUC: 0.667; *p* < 0.05) and indicated acute respiratory distress syndrome (ARDS) among septic patients (AUC: 0.686; *p* < 0.05). Furthermore, a combination of Flii and other sepsis markers seemed to offer an improved diagnostic performance (sepsis vs. non-sepsis, AUC of Flii + gelsolin (GSN) + Gc-globulin + procalcitonin: 0.974; *p* < 0.001 and ARDS vs. non-ARDS, AUC of Flii + GSN + presepsin: 0.776; *p* < 0.001) compared with single markers even in the prediction of 14-day mortality (AUC of Flii + GSN + Gc-globulin: 0.76; *p* < 0.001). **Conclusions**: We adapted a properly precise and reproducible automated Western blot method to determine concentrations of Flii in human serum. Our results revealed the relationship between Flii and sepsis; however, Flii alone did not appear to be a prominent sepsis marker. When combined with other biomarkers, measurement of serum Flightless-I may provide additional value supporting patient care.

## 1. Introduction

Diagnosis and treatment of sepsis is considered one of the major healthcare challenges in the 21st century as it is responsible for the death of millions of patients worldwide in every year [1,2]. Efforts to reduce mortality include the early recognition of sepsis and sepsis-related organ failures, in which laboratory diagnostics play an essential role. Serum procalcitonin (PCT) and high-sensitivity C-reactive protein (hs-CRP) are still used as gold standard inflammatory parameters in the clinical evaluation of sepsis [3]. Apart from these, new markers for the diagnosis and prediction of sepsis have become the focus of recent research. In this context, several inflammatory biomarkers—such as interleukin-6 (IL-6), soluble triggering receptor expressed on myeloid cells 1 (sTREM-1), urokinase-type plasminogen activator receptor (uPAR), and the soluble CD14 receptor subtype (sCD14-ST or presepsin, PSEP)—are considered promising candidates [4,5,6]. Biomarkers of sepsis-associated organ dysfunction, including those for acute kidney injury (AKI), such as urinary tissue inhibitor of metalloproteinases-2 (TIMP-2) combined with insulin-like growth factor-binding protein 7 (IGFBP7) [7], as well as markers of immunosuppression, such as monocyte human leukocyte antigen–DR (mHLA-DR) [8], have also been investigated. However, these laboratory tests do not meet all current requirements. To improve the efficiency of the laboratory side, numerous sepsis biomarkers have been investigated recently but none of them have become part of the routine clinical practice [9,10].

Actin (MW = 42 kDa) is a ubiquitous protein which can be found not only in muscles but in every cell as an elementary component of the cytoskeleton (intracellular actin). It can appear in two main forms: globular (G) or monomeric and filamentous (F) or polymeric forms [11]. Actin—as a potential toxic compound in the peripheral blood—enters the circulation even under healthy conditions due to physiologic cell turnover. Therefore, as a homeostatic mechanism, proteins of an actin scavenger system are responsible for the elimination of released actin [12]. Besides that, the presence of actin has also been proven in the urine, which might serve as an alternative elimination pathway [13].

In sepsis, excessive pathological processes develop (pathogen-associated molecular patterns (PAMPs) and damage-associated molecular patterns (DAMPs)), which can cause multiple injuries in host cells as well. As a result of extensive cell damage, actin can be released in large amounts from the injured cells to the extracellular space and the bloodstream, which may lead to depletion of the scavenger capacity [14]. It has been noted that circulating free actin exhibits toxic effects: after spontaneous polymerization, F-actin can activate the coagulation system, leading to microthrombus formation and endothelial damage [15,16].

We have previously reported that serum gelsolin (GSN; MW = 83 kDa) as a major member of the actin scavenger system showed superior performance in differentiation of sepsis from the non-septic severe inflammatory state, and it might be a potential predictor of 14-day mortality in sepsis [17].

Another actin-binding protein called Flightless-I (Flii; MW = 145 kDa) belongs to the gelsolin superfamily and appears in intracellular and in secreted forms. Flii shows structural and functional similarity with GSN: it possesses six GSN homology domains (G1-6) with leucine-rich repeats (LRRs) and both Flii and GSN are capable of binding lipopolysaccharides (LPSs) besides actin. Flii has been described as an actin-remodeling protein with a significant role in cell motility and wound healing; furthermore, it may even be a modulator of inflammation owing to its interactions with the NLR-family pyrin domain containing-3 (NLRP3) inflammasome and the myeloid differentiation primary response protein 88–Toll-like receptor 4 (MyD88-TLR4) pathways [18,19].

The NLRP3 inflammasome is known as an important component in the host’s defense mechanisms against microbial pathogens in humans as well as in animals [20]. It has been suggested that Flii may function as a negative regulator of the NLRP3 inflammasome, thereby suppressing the secretion of IL-1β and the further pro-inflammatory activation. Flii binds pro-caspase-1 to prevent the formation of the NLRP3 inflammasome, whose mechanism is further enhanced by the binding of LRR Flightless-interaction protein-2 (LRRFIP2) to Flii. In addition, LRRFIP2 binding to NLRP3 also demonstrates an inhibitory effect on this pathway [21]. Concerning the TLR4 signaling pathway, in vitro investigations showed that Flii binds MyD88 to prevent the formation of TLR4-MYD88 complex, thereby diminishing NF-kB signaling in macrophages [19,22]. Besides the interactions with intracellular signaling, LPS binding of secreted Flii appears to inhibit macrophage stimulation, which can lead to a reduced cytokine secretion of macrophages.

The abovementioned interactions may have a protective effect against the overproduction of pro-inflammatory cytokines and the development of the cytokine storm [19,23]. The involvement of Flii in inflammatory signaling raises an association with the underlying processes in the development and course of sepsis (Figure S1).

The presence of Flii has already been demonstrated in plasma samples of healthy human individuals,; however, exact Flii levels were not reported [23,24]. Therefore, we aimed to determine the concentration of Flii in human serum samples and, moreover, to investigate serum Flii as a potential sepsis biomarker.

## 2. Materials and Methods

### 2.1. Patients and Studied Groups

Our study protocol was approved by the Regional Research Ethics Committee of the University of Pécs (no. 4327.316-2900/KK15/2011) in accordance with the Helsinki Declarations. All participants were fully informed, and written consent was obtained from all of them or their next of kin. Patients were enrolled in our single-center follow-up study between September 2017 and February 2019 at the Department of Anesthesiology and Intensive Therapy, Medical School, University of Pécs, Hungary. Blood samples of septic and non-septic patients included in our study were derived from the same biobank, which has been previously investigated by our research group [25]. From these collected and archived sera, we carried out our analysis retrospectively.

From patients with clinically confirmed sepsis, blood samples were obtained on the day of admission and on two further consecutive days during intensive care unit (ICU) treatment. Diagnosis of sepsis was established according to the Sepsis-3 definitions [9]. Exclusion criteria were age under 18 years, need for chronic dialysis, end-stage malignant disease, or the lack/withdrawal of consent. Septic patients were further categorized based on organ failures. Patients were considered to have moderate or severe acute respiratory distress syndrome (ARDS) when they exhibited a Horowitz index ≤200 mmHg with a positive end-expiratory pressure ≥5 cm H_2_O, following the Berlin definition [26]. Acute kidney injury (AKI) was established when elevated serum creatinine level (≥1.5-fold increase from the baseline in the last 7 days or ≥26.5 μmol/L increase within 48 h) or decreased urine output (<0.5 mL/kg/h for 6 h) was detected [27]. Septic shock was defined as the presence of persisting hypotension (requiring vasopressors to maintain MAP ≥65 mmHg) with plasma lactate levels >2 mmol/L despite adequate volume resuscitation.

Moreover, septic patients were divided into 2 groups according to the change in their condition during follow-up. Patients having a Sequential Organ Failure Assessment (SOFA) score at least 2 points lower on the 3rd day compared with the admission value were considered to have an improving condition.

Patients who underwent extended abdominal surgery (with or without thoracic involvement) were involved in the non-septic severe inflammation group with no documented or suspected infection. From non-septic patients, single blood samples were drawn on the first postoperative morning.

Healthy workers at our university served as controls (healthy state was assessed based on physical examination and on medical records). Volunteers with symptoms of acute inflammatory disorders or suffering from any chronic diseases (or having hs-CRP >5 mg/L) were excluded.

### 2.2. Sampling

Venous blood was collected in plastic tubes (native or anticoagulated with EDTA) using a closed blood sampling system (BD Vacutainer^®^, Franklin Lakes, NJ, USA). After centrifugation of clotted blood samples (10 min, 1500× *g*), serum specimens were stored in aliquots at −80 °C until analyses. Anticoagulated samples for blood count were analyzed immediately.

### 2.3. Determination of Serum Flii Levels

To determine serum Flii levels, the capillary electrophoresis-based Simple Western™ system (12–230 kDa Separation Module) of Protein Simple© (Bio-Techne, San Jose, CA, USA) was used with the “Wes” chemiluminescent detection method. The densitometric evaluation of chemiluminescence was carried out by Compass Software, Version 5.0.1 provided with the instrument [28]. The assay was performed according to the manufacturer’s instructions. Briefly, after thawing, serum samples were diluted 20-fold with the electrophoresis sample buffer containing β-mercaptoethanol and then were combined with the Fluorescent Master Mix in a ratio of 4:1 and heated at 95 °C for 5 min. A polyclonal anti-Flightless-I primary antibody (1:50 dilution; Rabbit Anti-Human Flightless-I, ref.no: A301-565A, Bethyl Lab. Inc. Montgomery, TX, USA) and a horseradish peroxidase (HRP)-labeled secondary antibody (Goat Anti-Rabbit IgG, included in the assay kit) were applied. In every run, the same pooled serum sample was also loaded in duplicates as the internal standard, which was pretreated identically with the samples.

**Figure 1:**
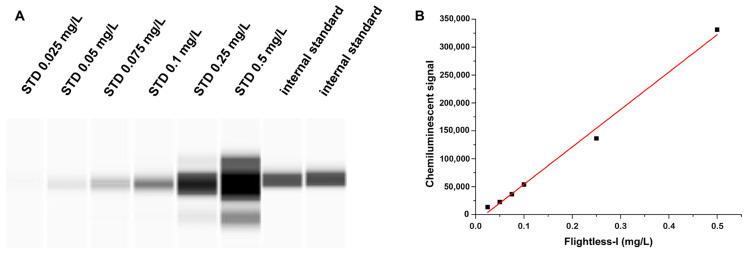
Calibration of the Flightless-I assay by using 6 different dilutions of the purified human Flii as the stock solution: (**A**) chemiluminescent signal of Flii standard series and the internal standard; (**B**) calibration curve of Flii in the range of 0.025–0.5 mg/L; R^2^ = 0.9932. STD: standard.

To calibrate the assay, purified recombinant human Flii expressed in Escherichia coli obtained from the Department of Biophysics, University of Pécs, Medical School, Hungary, was used [18]. Dilution series of the Flii standard of known concentration along with the internal standard were loaded in a separate run to establish a calibration line and to quantify the Flii concentration of the internal standard (Figure 1A). Afterwards, within each run, patients’ Flii levels were calculated by using the chemiluminescent signal ratios: samples’ luminescence referred to that of the internal standard.

### 2.4. Determination of Other Laboratory Parameters

Serum hs-CRP, PCT, urea, creatinine, total protein, albumin levels and complete blood count were measured using routine procedures at our accredited laboratory (Department of Laboratory Medicine University of Pécs, Hungary; accreditation no.: NAH-9-0008/2021). Serum GSN and Gc-globulin (Gc) levels were determined by our formerly described automated immune turbidimetric assays [17,29]. Plasma presepsin (PSEP) concentrations were measured by an automated quantitative Point of Care test (PATHFAST; LSI Medience Corporation, Tokyo, Japan).

### 2.5. Statistical Analysis

Statistical analysis was performed by IBM SPSS Statistics for Windows, Version 22 (IBM Corporation, Armonk, NY, USA). Since our quantitative data showed a non-normal distribution assessed by Kolmogorov–Smirnov or Shapiro–Wilk analyses, non-parametric statistical tests were used. To investigate differences between groups, Kruskal–Wallis, Mann–Whitney U, and chi-square tests were applied. Friedman’s analysis was used to compare data of different days during follow-up. Evaluation of relationships between continuous variables was performed by Spearman’s rank correlation test. Diagnostic or prognostic performance was analyzed by receiver operating characteristic (ROC) curves and areas under the curves (AUCs) were determined. Combinatory marker analysis was conducted by binary logistic regression. Model fit was assessed using the Hosmer–Lemeshow test, and multicollinearity was checked via Variance Inflation Factors (VIFs). The obtained probabilities for combined markers were used as test variables in ROC analysis. Values of *p* < 0.05 were considered statistically significant.

## 3. Results

### 3.1. Quantification of Flii by the Simple Western™ System

To quantify the Flii assay, six different dilutions of the purified human Flii as stock solution (Flii concentration: 755 mg/L) were applied, which covered the range of 0.025–0.5 mg/L. Therefore, the chemiluminescent signal of the (pre-diluted) internal standard was found within the calibrated range and its concentration was determined from the equation of the obtained calibration line (R^2^ = 0.9932) by considering the dilution factor (Figure 1B).

### 3.2. Demographic, Clinical, and Laboratory Data of Patient Groups

Demographic, clinical, and laboratory parameters of the participating patients are reported in Table 1. Septic and non-septic patients with severe inflammation were comparable in age and sex; however, controls were significantly younger. Regarding comorbidities, the frequency of cancer was higher in the non-sepsis group. Infective microorganisms were identified from hemocultures or other sources in 69% of septic patients. In most cases, Gram-positive (19%) or Gram-negative bacteria (12.5%) were detected; however, fungal (8%) and combined (29.5%) infections were also present. Conventional inflammatory markers (hs-CRP, PCT, white blood cell count (WBC)) were significantly elevated in sepsis while platelets (PLT) showed a lower count compared with non-septic patients. Major proteins of the actin scavenger system (GSN, Gc) exhibited decreased serum levels in patients with severe inflammation compared with controls and a further fall was observed in sepsis.

### 3.3. Serum Levels of Flii and Its Diagnostic Performance in Sepsis and in Sepsis-Related Organ Dysfunctions

**Figure 2:**
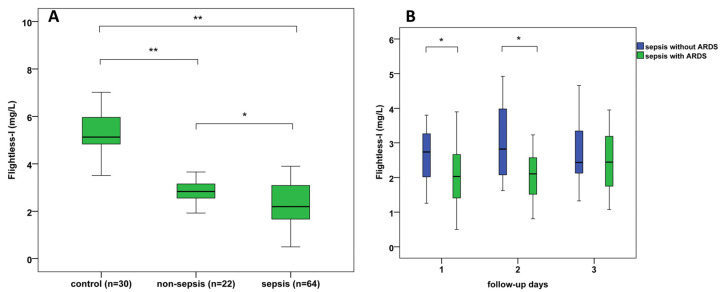
(**A**) Admission serum Flightless-I levels in septic, non-septic, and control patients; (**B**) 3-day follow-up of serum Flightless-I levels where septic patients are divided into those with ARDS and those without ARDS; * *p* < 0.05, ** *p* < 0.01. ARDS: acute respiratory distress syndrome.

In human serum, Flii was found in considerably lower amounts than GSN or Gc. Flii levels of healthy controls varied between 3.5 and 8.8 mg/L. Serum Flii concentrations were found to be lowest in the sepsis group, significantly higher Flii levels were detected in non-septic patients compared with septic cases (*p* = 0.021), and the highest values were observed in controls (*p* < 0.001) (Table 1, Figure 2A).

Regarding sepsis-related organ dysfunctions, 36 of 64 septic patients exhibited moderate or severe ARDS, 45 septic patients were diagnosed with AKI, and septic shock occurred in 28 cases.

We found significantly lower Flii levels in sepsis-related ARDS compared with septic patients without ARDS at admission (*p* = 0.011) and on the 2nd day of ICU treatment (*p* = 0.014) (Figure 2B). Furthermore, GSN, PSEP levels, and SOFA scores also showed differences (*p* < 0.05) between septic patients with and without ARDS (Table 2). However, admission serum albumin, hs-CRP, PCT, Gc levels, WBC, and PLT counts did not differ significantly between these patients’ groups.

Flii levels did not show significant differences in sepsis-related AKI (2.47 (1.60–3.14) mg/L) compared with septic patients without AKI (1.97 (1.73–2.67) mg/L) nor in septic shock (2.39 (1.58–2.91) mg/L) compared with septic patients without shock (2.08 (1.71–3.22) mg/L).

### 3.4. Investigating 14-Day Mortality in Sepsis

Within 14 days, app. 41% of ICU patients did not survive the septic episode. First-day SOFA scores proved to be significantly (*p* < 0.01) higher, whereas serum albumin levels were significantly lower (*p* < 0.05) in non-survivors than in survivors. Investigating first-day serum Flii levels regarding 14-day mortality, similar concentrations were found among survivor and non-survivor patients. In contrast to Flii, GSN levels were significantly lower in non-survivors compared with survivors on the day of admission (*p* = 0.01) (Table 3). There was no significant difference between survivors and non-survivors regarding PCT, hs-CRP, PSEP, Gc levels, WBC, and PLT counts. During follow-up, no significant changes were observed in the kinetics of serum Flii levels among survivors and non-survivors. Similarly, non-significant trends were seen among septic patients with and without improving conditions.

### 3.5. Assessing Potential Diagnostic and Predictive Power of the Markers Studied by ROC Analyses

**Figure 3:**
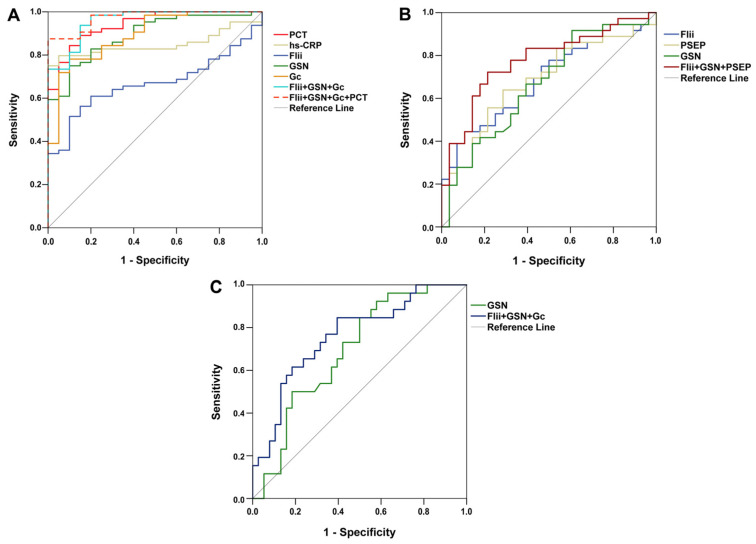
Receiver operating characteristic curves of first-day laboratory parameters for (**A**) differentiating sepsis from non-sepsis; (**B**) indicating acute respiratory distress syndrome among septic patients; and (**C**) predicting 14-day mortality in sepsis. Flii: Flightless-I; Gc: Gc-globulin; GSN: gelsolin; hs-CRP: high-sensitivity C-reactive protein; PCT: procalcitonin; PSEP: presepsin.

ROC analysis revealed that in the differentiation between septic and non-septic patients, first-day PCT and hs-CRP had significant diagnostic capacities. Besides them, GSN, Gc, and Flii also had significant diagnostic values (Figure 3A). In addition, after combined marker analysis, the combination of Flii, GSN, and Gc and that of Flii, GSN, Gc, and PCT were also proved to have significant diagnostic performances.

Among the laboratory parameters investigated, only Flii, GSN, and PSEP had significant diagnostic power to indicate ARDS (Figure 3B). Combined marker analysis showed that the combined diagnostic value of Flii, GSN, and PSEP was also significant.

The predictive power of our studied markers regarding 14-day mortality was also investigated. As a single marker, only first-day GSN had predictive capacity for the differentiation between survivors and non-survivors. On the other hand, the combination of the three actin-binding proteins (Flii, GSN, Gc) seemed to enhance the predictive value (Figure 3C). In Table 4, detailed results regarding ROC analyses are depicted.

### 3.6. Spearman’s Correlation Results

Moderate correlations were found between Flii and GSN (ρ = 0.557, *p* < 0.001), Gc (ρ = 0.341, *p* < 0.001), total protein (ρ = 0.675, *p* < 0.001), and albumin (ρ = 0.501, *p* < 0.001) and a weak relationship was seen between Flii and platelet count (ρ = 0.232, *p* = 0.001)). Flii showed moderate or weak inverse correlations with hs-CRP (ρ = −0.427, *p* < 0.001), PSEP (ρ = −0.289, *p* < 0.001), PCT (ρ = −0.187, *p* = 0.01), and WBC (ρ = −0.169, *p* = 0.012). Further associations were observed between SOFA score and several laboratory parameters (Table S1).

## 4. Discussion

Biomarker research has an outstanding potential to improve the efficiency of diagnostics and follow-up in sepsis [3,30]. In the present study, we aimed to investigate the performance of Flii as a possible novel marker of sepsis.

To our best knowledge, there have been no published data on the concentration of Flii in human serum. Our study seems to be the first giving a quantitative approach of Flii in human sera of healthy control individuals and in different sepsis conditions. In earlier research, Flii was measured by the classical Western blot technique from wound drainage and plasma, but the authors gave the luminescence intensities only instead of real concentrations [24].

Moreover, Flii from mouse plasma was measured with an in-house ELISA method but without standards and calibration [31]. The Simple Western™ system used in our study is suitable for quantitative determination of Flii from various matrices (serum, plasma, culture medium, and cell lysate as well). A formal validation of the assay’s precision was not performed in this study and represents an important goal for future work to confirm its reliability for clinical applications. However, compared to the classical Western blot, the automated method seems to be more precise because of its less user-dependency, it has a higher sample analyzing capacity (24 samples/run), and results can be acquired within 4 h. We were the first to obtain the serum Flii levels of healthy individuals. Although the number of controls was only 30, the measured protein levels could serve as exemplary data for the assessment of Flii concentration in healthy persons.

To our best knowledge, our study is the first to quantify Flii in septic patients. We found significantly decreased concentrations both in sepsis and in non-septic severe inflammatory conditions. Ruzehaji et al. studied plasma Flii in patients undergoing abdominal plastic operation and found decreased Flii levels after the surgery, but the change was not significant [24]. In our work, we obtained similar results in patients after abdominal surgery, but our data differed significantly from those of the controls. In septic patients, the decrease in Flii was even higher and more significant compared with the results for non-septic inflammatory cases. However, the diagnostic performance of the Flii test was less than that of GSN and Gc-globulin determinations as assessed by ROC analysis.

The mechanism of the decrease in Flii concentrations seen in sepsis is not elucidated yet. It is plausible that the reduction in serum Flii level may be explained by its increased elimination caused by the Flii’s scavenger function. Besides GSN and Gc, secreted Flii can also be a component of the actin scavenger system, whose role is to prevent toxic effects of free actin by eliminating it from plasma. Numerous studies suggested that actin-bound GSN and Gc in the blood are cleared by the reticuloendothelial system; therefore, the levels of both proteins rapidly decrease [12,14,17]. Furthermore, Flii has the ability to bind LPS too. It has been supposed that secreted Flii may act as a scavenger by binding and neutralizing LPS, thereby limiting the immune response [23]. However, exact elimination pathways of Flii are not known; it might be related to the reticulo-endothelial system and proteolytic degradation as well.

The intracellular form of Flii may also be involved in the modulation of inflammatory processes by influencing NLRP3 and MyD88-TLR4 pathways [19]. Based on its roles in the inflammatory signaling, we hypothesize that Flii may act as a protective factor in patients with sepsis by the suppression of cytokine release and the inhibition of immune overactivation. Reduced serum Flii in sepsis might even enhance the worsening of sepsis, although the serum Flii value only refers to the amount of the extracellular form.

It has been reported that Flii negatively affects wound healing. In mice with Flii deficiency, a faster wound repair was noted while Flii overexpression seemed to reduce proliferation of dermal fibroblasts, with epithelial migration and expression of tight junction proteins (e.g., Claudin-1, ZO-2) causing a delayed wound closure [19,32]. However, increased Flii expression of the injured skin was observed compared with the intact condition, which might serve to limit cytokine production and to demarcate inflammation [23].

Furthermore, it has been shown that the interaction between the LRR of Flii and the monomeric GTPase R-Ras is essential for cell adhesion and the growth of cell extensions enabling cell migration [33].

It is already known that Flii is produced by fibroblasts and macrophages. In cell culture models, it is also shown that the secretion of Flii increases in cellular injuries and in excess LPS exposure [23]. Among sepsis-related organ failures, only the development of ARDS could be predicted by Flii determination. Recently, the decrease in GSN levels has been described in ARDS, which was also found in our study together with low Flii levels when compared with the corresponding values in septic patients without ARDS [25,34].

We could not find any change in the concentration of Flii in septic patients during the occurrence of AKI or septic shock.

The organ-specific relationship between Flii and the condition of lungs is unknown. Our findings regarding the diagnostic capacity of Flii in sepsis-related ARDS are only preliminary and must be confirmed by further investigation involving a higher number of patients.

In our study, Flii did not show any predictive value regarding the 14-day mortality of sepsis. GSN proved to be the only predictor among the examined lab parameters to foretell mortality, similarly to our previous study [17].

In addition, several combined marker analyses revealed that the combination of actin-binding proteins together with classical parameters could enhance their diagnostic/predictive power when compared to single biomarkers. Furthermore, while the elevation in the classical gold standard sepsis marker, procalcitonin, is primarily associated with bacterial sepsis, actin-binding proteins including Flightless-I indicate a systemic cell damage independent of the pathogen. Diagnosis of sepsis and its related organ dysfunctions, such as ARDS, is largely based on alterations in clinical physiological parameters (e.g., the variables included in the SOFA score), which also hold prognostic value; for instance, a low Horowitz index is associated with a higher risk of mortality [26]. More recently, changes in additional clinical parameters, such as PaCO_2_, have also been linked to adverse outcomes in septic patients [35]. Given the observed decline in se-Flii levels in sepsis and sepsis-related ARDS, it may be worthwhile developing a multiparametric approach that integrates both clinical variable assessment and biomarker measurements (e.g., Flii and other markers [PCT, GSN, Gc]) to potentially enhance the diagnostic and predictive performance of standard clinical variables.

Flii showed a medium correlation with serum total protein and albumin levels, indicating that in sepsis, the loss of non-specific proteins might also be responsible for the decreased Flii concentrations. Flii showed the strongest correlation with GSN among the major actin binding proteins. It might be explained by its similar structure to GSN and their common properties to bind actin and LPS simultaneously.

During the 3-day follow-up, Flii levels did not change despite the improvement in the septic patients’ status as verified by the decrease in their SOFA scores. It seems that re-normalization of the Flii concentrations requires a longer time scale after the healing process. Similarly, GSN did not alter within the follow-up period.

Our study carries some limitations. First, we have not analyzed other organ failures, and the patient number should be increased in the future for better understanding of the role and diagnostic potential of Flii in sepsis. Moreover, it cannot be excluded that other critical conditions than sepsis (e.g., malignancies) might have an influence on the Flii levels. It must also be mentioned that our control individuals were younger than the septic patients,; however, other studies reported a similar discrepancy. Investigation of Flii levels also in an older healthy population would be required to clarify the potential influence of age.

Although the automated Western blot method performs better than the classical version, it cannot be incorporated in the ICU management. In the case, we could verify our results in a larger patient cohort in the future, and development of a rapid point-of-care test for Flii measurement should be established. This test, potentially in a multi-marker panel, could allow integration of Flii with clinical scores like SOFA at the bedside, facilitating earlier risk stratification.

## 5. Conclusions

In the present study, we have quantified the serum levels of Flightless-I in control and in septic patients by an automated Western blot system, which seems to be more precise and reproducible with significantly higher throughput than the classical Western blot. Our results undoubtedly demonstrated the association of Flii with sepsis; however, Flii alone did not appear to be a prominent sepsis marker. When combined with other biomarkers, measurement of serum Flightless-I could offer incremental value supporting patient care. Further research may help clarify the role of Flii in the modulation of immune response. The Simple Western™ system could serve as an innovative method for the first-line quantitative investigation of serum proteins in various diseases.

## Figures and Tables

**Table 1:** Patients’ characteristics and admission laboratory data.

	Control (n = 30)	Non-Sepsis (n = 22)	Sepsis (n = 64)	*p* Value
Clinical data				
Age, years	36 (28–42)	65 (58–75)	68 (57–73)	<0.001 ^a,b^
Males, n (%)	15 (50)	13 (59)	42 (66)	n.s.
CVD, n (%)	-	17 (77.3)	50 (78.1)	n.s.
COPD, n (%)	-	0	12 (18.8)	0.029
CKD, n (%)	-	0	8 (12.5)	n.s.
Type II DM, n (%)	-	4 (18.2)	18 (28.1)	n.s.
Immunological diseases, n (%)	-	1 (4.5)	2 (3.1)	n.s.
Malignancy, n (%)	-	19 (86.4)	18 (28.1)	<0.001
First-day parameters:				
SOFA score	-	-	10 (8–12)	-
hs-CRP, mg/L	0.8 (0.4–1.7)	109.9 (89.5–122.7)	284.6 (165.1–384.4)	<0.001 ^a,b,c^
PCT, ng/mL	-	0.8 (0.2–2.2)	11.0 (4.5–49.9)	<0.001
WBC, G/L	6.6 (5.7–7.7)	10.4 (8.6–11.9)	16.5 (10.6–22.8)	<0.001 ^a,b,c^
PLT, G/L	279.5 (250.0–304.0)	251.5 (196.3–340.8)	199.0 (140.3–300.5)	0.005 ^b,c^
se-GSN, mg/L	82.9 (76.2–88.5)	39.1 (24.8–43.5)	11.2 (6.1–21.0)	<0.001 ^a,b,c^
se-Gc, mg/L	406.8 (370.2–444.3)	323.1 (275.8–374.7)	233.9 (178.1–268.9)	<0.001 ^a,b,c^
se-Flii, mg/L	5.12 (4.77–5.96)	2.83 (2.53–3.17)	2.20 (1.65–3.11)	<0.001 ^a,b,c^

Data are expressed as number (%) or median (IQR). Microbiological findings are presented by numbers. Superscript lowercase letters show post hoc analysis. ^a^: control—non-sepsis, *p* < 0.001; ^b^: control—sepsis, *p* < 0.005; ^c^: sepsis—non-sepsis, *p* < 0.05. CKD: chronic kidney disease; COPD: chronic obstructive pulmonary disease; CVD: cardiovascular disease; Flii: Flightless-I; Gc: Gc-globulin; GSN: gelsolin; hs-CRP: high-sensitivity C-reactive protein; n.s.: non-significant; PCT: procalcitonin; PLT: platelet; SOFA score: Sequential Organ Failure Assessment Score; WBC: white blood cell.

**Table 2:** Admission parameters of septic patients with and without ARDS.

	Sepsis Without ARDS (n = 28)	Sepsis with ARDS (n = 36)	*p* Value
First-day parameters:			
SOFA score	8 (7–10)	11 (10–13)	<0.001
se-albumin, g/L	23.4 (19.6–28.8)	23.9 (19.6–27.6)	n.s.
hs-CRP, mg/L	289.8 (157.1–360.3)	280.5 (194.0–390.1)	n.s.
PCT, ng/mL	8.2 (3.3–33.8)	12.8 (7.1–60.4)	n.s.
PSEP, pg/mL	683.5 (477.3–2688.5)	1779.0 (631.8–4252.5)	0.011
WBC, G/L	17.7 (14.3–22.1)	13.1 (10.1–23.1)	n.s.
PLT, G/L	232.5 (156.8–297.8)	174.0 (112.3–319.8)	n.s.
se-Gc, mg/L	238.6 (178.1–259.5)	222.7 (177.6–270.6)	n.s.
se-GSN, mg/L	16.1 (8.5–30.5)	9.8 (5.2–17.9)	0.021
se-Flii, mg/L	2.74 (1.99–3.28)	2.03 (1.36–2.71)	0.011

Data are expressed as median (IQR). ARDS: acute respiratory distress syndrome; Flii: Flightless-I; Gc: Gc-globulin; GSN: gelsolin; hs-CRP: high-sensitivity C-reactive protein; n.s.: non-significant; PCT: procalcitonin; PLT: platelet; PSEP: presepsin; SOFA score: Sequential Organ Failure Assessment Score; WBC: white blood cell.

**Table 3:** Admission parameters of survivor and non-survivor septic patients regarding 14-day mortality.

	Survivors (n = 38)	Non-Survivors (n = 26)	*p* Value
First-day parameters:			
SOFA score	9 (7–11)	11 (10–13)	0.006
se-albumin, g/L	24.2 (21.4–28.7)	20.0 (17.1–25.3)	0.023
hs-CRP, mg/L	287.2 (157.0–352.3)	280.5 (193.7–397.7)	n.s.
PCT, ng/mL	11.5 (3.7–53.3)	10.8 (7.3–34.7)	n.s.
PSEP, pg/mL	935.5 (419.5–1588.5)	1281.0 (590.0–3214.5)	n.s.
WBC, G/L	17.1 (10.6–25.9)	14.4 (10.6–20.4)	n.s.
PLT, G/L	192.0 (139.3–287.3)	220.0 (137.8–328.3)	n.s.
se-Gc, mg/L	238.6 (179.5–276.3)	222.0 (161.8–268.8)	n.s.
se-GSN, mg/L	15.8 (8.5–27.9)	8.4 (5.4–15.5)	0.01
se-Flii, mg/L	2.10 (1.73–3.21)	2.39 (1.55–2.93)	n.s.

Data are expressed as median (IQR). Flii: Flightless-I; Gc: Gc-globulin; GSN: gelsolin; hs-CRP: high-sensitivity C-reactive protein; n.s.: non-significant; PCT: procalcitonin; PLT: platelet; PSEP: presepsin; SOFA score: Sequential Organ Failure Assessment Score; WBC: white blood cell.

**Table 4:** Detailed results of the ROC analyses.

Differential Diagnosis	Parameter	ROC AUC (95% CI)	*p* Value
Sepsis vs. non-sepsis	PCT	0.941 (0.890–0.991)	<0.001
	hs-CRP	0.849 (0.768–0.930)	<0.001
	Flii	0.667 (0.554–0.780)	<0.05
	GSN	0.892 (0.821–0.963)	<0.001
	Gc	0.893 (0.816–0.970)	<0.001
	Flii + GSN + Gc	0.959 (0.916–1.0)	<0.001
	Flii + GSN + Gc + PCT	0.974 (0.946–1.0)	<0.001
ARDS vs. non-ARDS	PSEP	0.687 (0.556–0.817)	<0.05
	Flii	0.686 (0.556–0.815)	<0.05
	GSN	0.669 (0.535–0.804)	<0.05
	Flii + GSN + PSEP	0.776 (0.661–0.891)	<0.001
14-day mortality: yes/no	GSN	0.690 (0.561–0.819)	<0.05
	Flii + GSN + Gc	0.760 (0.640–0.880)	<0.001

ARDS: acute respiratory distress syndrome; CI: confidence interval; Flii: Flightless-I; Gc: Gc-globulin; GSN: gelsolin; hs-CRP: high-sensitivity C-reactive protein; PCT: procalcitonin; ROC AUC: receiver operating characteristics area under the curve; PSEP: presepsin.

## Data Availability

The manuscript includes the anonymized dataset of patients as electronic Supplementary Material.

## Supplementary Materials

The following supporting information can be downloaded at https://www.mdpi.com/article/10.3390/biomedicines13122850/s1, Figure S1. The main roles of Flightless-I and its associations with sepsis. (Images were derived from Servier Medical Art (https://smart.servier.com); NIAID NIH BioArt Source; Paulos, C.M. et al. [36], https://www.genecards.org/cgi-bin/carddisp.pl?gene=FLII (accessed on 14 November 2025)). Table S1. Correlations of SOFA score with the measured laboratory parameters in septic patients.
