# Supplementary material for: Quantification of the Actin-Binding Protein Flightless-I in Human Serum by Automated Western Blot System and Investigation of Its Diagnostic Potential in Sepsis
Source: Biomedicines. 2025 Nov 21;13(12):2850. doi: 10.3390/biomedicines13122850 (PMC12730203; doi:10.3390/biomedicines13122850)
Supplement: Supplementary file 1 [file biomedicines-13-02850-s001.zip › Table S1.pdf]

Table S1. Correlations of SOFA score with the measured laboratory parameters in septic patients.

SOFA score	Spearman’s rho	p value
hs-CRP	0.303	<0.001
PCT	0.383	<0.001
se-urea	0.363	<0.001
se-creatinine	0.475	<0.001
se-total protein	-0.146	n.s.
se-albumin	-0.154	0.046
WBC	0.210	0.006
PLT	-0.274	<0.001
PSEP	0.619	<0.001
se-Gc	-0.393	<0.001
se-GSN	-0.232	0.002
se-Flii	-0.199	0.010

Flii: Flightless-I; Gc: Gc-globulin; GSN: gelsolin; hs-CRP: high sensitivity C-reactive protein; n.s.: non-significant; PCT: procalcitonin; PLT: platelet; PSEP: presepsin; SOFA score: Sequential Organ Failure Assessment Score; WBC: white blood cell.
