# Supplementary figures and images for: Quantification of the Actin-Binding Protein Flightless-I in Human Serum by Automated Western Blot System and Investigation of Its Diagnostic Potential in Sepsis
Source: Biomedicines. 2025 Nov 21;13(12):2850. doi: 10.3390/biomedicines13122850 (PMC12730203; doi:10.3390/biomedicines13122850)

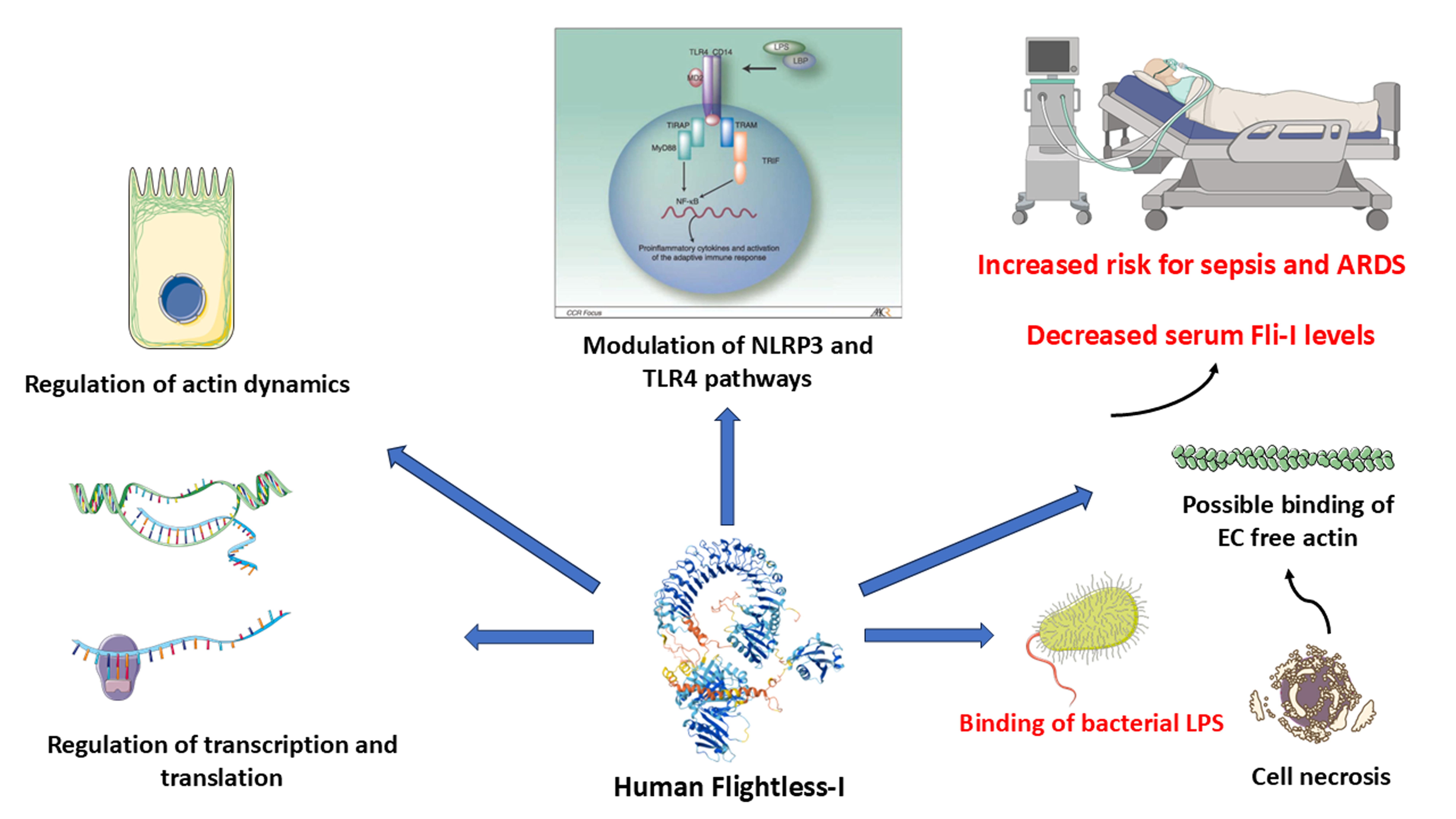

Supplement: Supplementary file 1 [file biomedicines-13-02850-s001.zip › Figure S1 Roles of human Flightless-I.tif]
